# The Ethical Assessment of the Stay-At-Home Order in South Africa in Light of The Universal Declaration of Bioethics And Human Rights (UNESCO)

**DOI:** 10.1007/s11673-023-10304-0

**Published:** 2023-10-26

**Authors:** A. L. Rheeder

**Affiliations:** Faculty of Theology, North-West University, Potchefstroom Campus, Private Bag X 6001, Potchefstroom, 2520 South Africa

**Keywords:** Global bioethics, Universal declaration of bioethics, Autonomy, Justice, COVID-19 pandemic

## Abstract

The South African government announced the much-discussed stay-at-home order between March 27 and April 30, 2020, during what was known as lockdown level 5, which meant that citizens were not allowed to leave their homes. The objective of this study is to assess the stay-at-home order against the global principles of the UDBHR. It is deducible that, in reference to the UDBHR, the government possessed the right to curtail individual liberty, thereby not infringing on Article 5 of the UDBHR and therefore, in this context, passes the test of the UDBHR. However, it remains uncertain at present whether the limitation of freedom imposed by the South African stay-at-home order was successful in controlling the spread of COVID-19 and protecting individuals from harm. Initial investigations also indicate that individuals who are particularly vulnerable may not have received equitable treatment in accordance with the principle outlined in Article 10, therefore, it can be cautiously and modestly argued that the stay-at-home order does not withstand scrutiny when assessed against the UDBHR. Given the continued discussion about the efficacy of limiting freedom to control the spread of COVID-19, and the growing conviction that the advancement of justice is being called into question, the notion of least restriction ought to be considered seriously. Ten Have (2022) is correct in asserting that global bioethics should also seriously consider other principles beyond an almost exclusive focus on limiting individual freedom. The preliminary conclusion is that the potential implementation of the stay-at-home order in the future must be seriously reconsidered.

## Introduction

The World Health Organization (WHO) declared COVID-19 to be a global pandemic on March 11, 2020. After the outbreak of the virus but before the availability of a vaccine (and even after that), governments were faced with the question as to how to effectively limit the spread of the virus. On the March 5, 2020, South Africa’s first COVID-19 infection was reported (Heywood 2020). Like several countries in the world, the South African government also introduced forced confinement or a state of lockdown. The present article brackets the containment period between March 27 and April 30, 2020 (level 5) and will focus on the fact that citizens were not allowed to leave their home or yard, also known as the so-called “stay-at-home order” (Adebiyi, et al. 2021; Human Rights Watch 2020). This measure was introduced under the Disaster Management Act (Heywood 2020).

Heywood (2020) claims that this order without doubt affected the constitutional rights of the citizens. The research question of this article is whether the order, as enforced during the lockdown, passes the test of the Universal Declaration of Bioethics and Human Rights (UDBHR) of The United Nations Educational, Scientific and Cultural Organization (UNESCO). The aim of the article is to ethically assess the practice of staying at home in South Africa in the light of the UDBHR. The stay-at-home order will be assessed in terms of articles 5 and 10 of the UDBHR. Article 5 deals with the global principle of autonomy and personal responsibility and article 10 with justice.

The main theoretical assertion of the article posits that the implementation of the stay-at-home order does not violate Article 5 of the UDBHR. However, it raises concerns regarding the extent to which the directive truly upholds principles of justice as outlined in Article 10.

Why focus on the UDBHR? It has been part of a larger research project for the past eleven years. In 2005, all the member states of UNESCO accepted the UDBHR. The adoption of this document is a remarkable achievement in the sense that it is the first and currently the only bioethical political (and human rights) instrument that has been unanimously adopted by all the governments of the world, including South Africa (UNESCO 2005). The innovative dimension of the instrument is that it morally commits all states of the world to taking its principles seriously and promote them in their countries (Ten Have 2017). Although the text is not enforceable, countries and scientists are called upon by the UDBHR to make it part of all bioethical debates (art. 19, 23; UNESCO 2006). The UDBHR moreover forms a unique global instrument for assessing all bioethical matters. Without the voice of this instrument, the bioethical debate is not complete. It must also be recognized that it is always easier to write critically about any matter in retrospect and that the government had to act quickly (Kraaijeveld 2021). However, moral issues and problems that were created by the forced confinement cannot simply be ignored or dismissed as unimportant: they deserve discussion and a global overview, as will be demonstrated here.

Subsequently, attention will be devoted to the concept of forced confinement (stay-at-home order), as well as forced confinement in relation to Articles 5 and 10 of the UDBHR.

## Forced Confinement

After the outbreak of the COVID-19 virus in December 2019 in the city of Wuhan, the Chinese government set the first precedent by curtailing almost totally private and public life by confining people to their homes. In Europe, after the outbreak of COVID-19, Italy was the first country to follow in the footsteps of China (Kraaijeveld 2021;l Ten Have 2022). South Africa experienced various levels and forms of forced confinement for approximately two years. Enforced confinement (level 5) began at midnight on Thursday evening March 26, 2020 and was enforced for approximately three weeks. After two weeks of forced confinement, the president announced that forced confinement would be extended until April 30, 2020 (Kell et al. 2022).

Forced confinement is generally understood as the stay-at-home order, which entails that the individual is only allowed to leave his or her house for the most essential places such as the supermarket, pharmacy, doctor, or hospital (Smart et al. 2020). Citizens were not allowed to go outside their yard. So, jogging, cycling, or walking with dogs was illegal and those who did try to do so were prosecuted. Citizens were also not allowed to visit sick family or friends in a responsible manner. For about thirty-five days, citizens of South Africa were forced to stay in their homes and, where possible, their yard. Part of the containment measures was that, when individuals, according to their judgment, had to urgently leave home to travel outside the province, the act had to be justified by an official document from the court (Adebiyi, et al. 2021).

The main purpose of the forced confinement was the protection of defenceless people such as the elderly, those with underlying medical conditions, and persons whose immunity was suppressed such as patients receiving chemotherapy, as well as preventing the overwhelming of health systems (hospitals) and the further spread of the virus. The goal of forced confinement was generally described as “flattening the curve” (Kraaijeveld 2021; Heywood 2020) .

Forced confinement means that the decision to contain the virus is enforced by the power of the state so that certain acts are criminalized. Fines, detention, arrest, and, in some cases, imprisonment, will be applied to force citizens so as to coerce them into obeying the containment rules. The SA government deployed around 24,000 army members to ensure that people stayed inside their homes and yards. Within the first seven days of forced confinement, more than 2,000 people were arrested, while some citizens were assaulted (Human Rights Watch 2020). Heywood (2020) claims that approximately 400,000 citizens were arrested for various offenses and approximately twelve people lost their lives due to police brutality in the period of total containment. These people now have a criminal record even though many of the arrests were arbitrary in nature. Forced confinement is in fact one of the most extreme measures that a government can introduce because it places the greatest restriction on the autonomy, freedom, and responsibility of the individual (Smart, et al. 2020). It means that people are no longer free to act as they see fit. They are no longer free to decide for themselves about following the necessary measures for the sake of others. There is no freedom to do the right thing because the rights are enforced.

At its core, freedom of movement (and association and trade) is restricted by the stay-at-home order (Bohler-Muller, et al. 2020; Heywood 2020). It is a fundamental right that is strongly endorsed by the UDBHR (Articles 2c, 2d, 3, 11, 12). Article 28 states the following: “Nothing in this Declaration may be interpreted as implying for any State, group or person any claim to engage in any activity or to perform any act contrary to human rights, fundamental freedoms and human dignity.” The stay-at-home order affects not only article 5 of the UDBHR but also article 10, which deals with justice and is to be discussed later in the present argument.

## Autonomy

Article 5 of the UDBHR states principle of autonomy or freedom in global bioethics as follows: “The autonomy of persons to make decisions, while taking responsibility for those decisions and respecting the autonomy of others, is to be respected” (UNESCO 2006). Autonomy has always been considered to be an imperative bioethical and human rights principle that must be protected as far as possible. In bioethics, the principle of autonomy expresses the concept of freedom. In a negative sense, freedom indicates the absence of restrictions on how the individual thinks and acts while, in a positive sense, it indicates the exercise of self-decision-making. Freedom has always formed the heart of the human rights movement and is a core concept in democratic societies (Ten Have and Patrão Neves 2021a). Within the context of the UDBHR, freedom and responsibility are considered principles derived from human dignity as their foundation (art. 3; UNESCO 2006). The application of human freedom and responsibility expresses the dignity of the individual (Evans 2009).

However, this does not mean that there is such a thing as absolute freedom. Absolute freedom can simply be considered a utopia or an empty abstraction (Ten Have and Patrão Neves 2021a). That is why article 5 of the UDBHR connects freedom with responsibility (Patrão Neves 2016). The concept of responsibility in article 5 carries two meanings according to most commentators. The first meaning is that individuals possess the autonomy to freely make health decisions about themselves and take responsibility for those decisions, that is, they accept legal responsibility for these. Thus, a patient can refuse cancer treatment and must take responsibility for that decision. The second interpretation has a moral meaning in the sense of responsibility for others. In their discussion of article 5, this moral meaning is described by Snead and Mulder-Westrate (2014) : “It urges respect for autonomy but reminds the reader that individuals bear responsibility for the choices they make and must pay due regard for the good or others” (cf. also Evans 2009). This statement resonates with the following words included in article 5 of the UDBHR: “while taking responsibility for those decisions and respecting the autonomy of others.” In other words, autonomy always takes into account the interests and rights of others.

This moral understanding of responsibility in the UNESCO declaration is also strengthened by article 14 (Social responsibility and health), which indicates that one has a social responsibility to promote the health of others (Patrão Neves 2016). On the one hand, autonomy without responsibility ends in arbitrariness, which means that the person and his decisions do not take into account the interests of the other person. On the other, it is also true that, where freedom is absent, there can be no question of responsibility, so that the matter becomes one simply of hierarchical coercion (UNESCO 2008).

The question arises as to whether the limitation of autonomy/freedom is permissible according UDBHR? The UDBHR suggests that there is a possibility. Article 27 of the UDBHR states that:

If the application of the principles of this Declaration is to be limited it should be done by law, including laws made in the interests of public safety, around the investigation, detection and prosecution of criminal offences for the protection of public health or for the protection of the rights and freedoms of others. Any such law needs to be consistent with international human rights law. (UNESCO 2006)

It is evident that if there is a need to restrict people’s rights, it can be justified for the purpose of safeguarding public health, among other considerations. According to this principle, also known as the harm principle, people’s rights may be limited if these can harm the health of others (Robinson 2009). Article 14 of the UDBHR indicates that the State has the responsibility to promote the health of citizens (UNESCO 2006).

It has been demonstrated that the UDBHR allows for the possibility of restricting human freedom within the context of health. Individual liberty is not in conflict with the common good. The question arises as to whether this limitation of autonomy/freedom through the stay-at-home order has effectively limited the spread of COVID-19 and therefore was justified (Ten Have 2022).

On the one hand, certain researchers maintain the conviction that the implementation of the stay-at-home order in South Africa had negligible impact and argue that the safeguarding of autonomy/freedom would have been more effective in preventing the spread of COVID-19. On the other hand, alternative studies present contrasting findings. Never before in the annals of human history have such a significant portion of the global populace been mandated to remain confined to their homes for an extended duration (Ten Have 2022), therefore, it is possible to have differing opinions about the outcomes of the stay-at-home orders in the early stages following the end of the pandemic, necessitating further extensive research in this regard.

On the one hand, academic and international studies claim that, in South Africa, no decrease but rather an increase in daily new infections in the period between March 27 and the end of April 30 occurred (Ritchie, et al. 2022). According to Ritchie, et al., the infection rate rose from fifty to 247 per day. The WHO’s figures differ slightly from Ritchie, et al., as they point to an infection growth of 153 to 354 per day during the stay-at-home order (WHO 2022). If the order to stay indoors was at all effective, the infection rate should have been drastically reduced within the first seven to fourteen days but, according to a study done by Smart, et al., and others, this simply did not happen (Smart, et al. 2020; Alex, et al. 2020; Yamin 2021). This position appears to have been substantiated by studies conducted in China and Quebec. A study in China came to the following conclusion: “All identified outbreaks of three or more cases occurred in an indoor environment, which confirms that sharing indoor space is a major SARS-CoV-2 infection risk” (Qian, et al. 2021).

Given the aforementioned information, viz. the absence of a decline and, instead, an upward trend in daily new infections, some researchers are of the conviction that the radical stay-at-home order in South Africa did not make a considerable difference to the prevention of infection: on the contrary, it could have promoted it. This indicates that, when communities are confronted with an epidemic, the best way to counter it is to promote freedom where the least intrusion on social functioning is made. The global bioethical principle of freedom with responsibility would therefore not simply be an abstract concept without any value for human health. As the discussion above suggests, the recognition of freedom can contribute to the protection of people’s health. Heywood (2020) is also of the opinion that focus on autonomy could make a major contribution to the prevention of infection.

The argument proceeds by maintaining that promoting freedom would entail the government granting citizens the freedom to act responsibly in a manner that would prevent or at the very least mitigate the spread of the virus. Citizens’ freedom can be respected by allowing them to move, walk, or jog outside their yard as long as they maintain social distance from other people, which is not a complicated concept and has been applied by most people. Human freedom and responsibility can be extended not only to walking, jogging, or cycling outside the house but also, at least, the right to visit family and friends, even across provincial borders. They may even continue to visit them under certain circumstances and may continue to practice their profession under certain conditions. Respecting autonomy may be relatively small or limited but still be significant and contribute to civic health.

Promoting freedom and responsibility means that the government accepts that citizens are responsible and will engage proactively with them on this basis. A conversation with such citizens can take place through a speech by the president of a country, advertisements, and social media. The content of the conversation may involve the following three matters: a) passing on knowledge of what is needed to contain the spread the virus (stay-at-home as much as possible, use of masks, social distancing and so on); b) the explanation as to why these measures are necessary, with emphasis on promoting the interests of the other person, solidarity, social responsibility; c) explanation of the meaning of personal responsibility to comply with measures that have been introduced by the government. Good communication could play a major role in preventing infection (Heywood 2020). Respect for autonomy and responsibility does not mean that everything must remain as it was before the outbreak of such a virus. The general expectation is that governments should take action to limit the spread of the virus. For example, mass gatherings can be limited due to the high risk of spreading the virus because there is no alternative to accommodate mass people safely (Kraaijeveld 2021).

On the other hand, international research has emerged that presents divergent findings and challenges the aforementioned outcomes and beliefs by positing that the implementation of stay-at-home orders has substantially reduced the transmission of COVID-19 due to the limitation of personal freedoms. In support of this perspective, a retrospective cohort study conducted in South Africa demonstrated a 14.1 per cent reduction in the risk of COVID-19 transmission as a result of the stay-at-home order (Pillai et al. 2020). Moreover, several other studies conducted within the United States context provide further confirmation of the South African study’s findings, highlighting the effectiveness of stay-at-home orders in curbing the spread of COVID-19 (Fowler et al., 2021; Jiang, et al. 2022; Wang 2022)

The objective of this study is to assess the stay-at-home order against the global principles of the UDBHR. The stay-at-home order represents a clear infringement upon individual freedom, yet the UDBHR allows for the restriction of individual freedom to protect the individual’s health. It can be concluded that, when tested against the UDBRH, the government had the right to limit individual freedom and therefore did not violate Article 5 of the UDBHR. It can be argued that the purpose of the restriction was to promote the interests and well-being of the individual. However, there is no consensus as to whether the stay-at-home order in South Africa prevented the spread of COVID-19 and protected the individual from harm. Whether the drastic stay-at-home order was more effective than respecting autonomy/freedom remains (currently) uncertain.

## Justice

The UDBHR has accepted the concept of justice as a global bioethical principle in Article 10 (centred on equality, justice and equity) which is formulated as follows (Ten Have and Patrão Neves 2021b): “The fundamental equality of all human beings in dignity and rights is to be respected so that they are treated justly and equitably” (UNESCO 2006). The present discussion focuses on the idea that people “are to be treated justly.” Justice here means equal and fair treatment as well as the fair distribution of advantages and disadvantages (UNESCO 2008; Ten Have and Patrão Neves 2021b). Within the context of the UDBHR, justice is also considered to be derived from human dignity as its foundation (art. 3; UNESCO, 2006). The application of individual justice is to recognize and respect the human dignity of the person (D’Empaire 2009).

In the present subsection, the distribution of burdens related to COVID-19’s stay-at-home order is briefly examined. A variety of aspects of the stay-at-home order and injustice could be discussed, such as the prohibition that informal workers may not work (Fisher, et al. 2022), but the health aspects will be discussed here.

One might think that the stay-at-home order is eminently democratic: after all, everyone, rich and poor, young and old, must stay at home. Yet it is not that simple. The stay-at-home order may be unfair, because the burden of staying at home may be experienced disproportionately by poorer individuals and groups (Fisher, et al. 2022). There is the reality of unequal conditions at home (Yamin 2021; Ten Have 2022). Forced confinement can disproportionately harm defenceless communities (Ten Have 2022). Circumstances at the homes of citizens can differ drastically so that some residents may suffer more than others. For most persons, it is difficult to stay inside the house for long periods, but this measure can place a much greater burden on people in the lower socio-economic strata than on those who enjoy a higher economic status (Kell, et al. 2022)

People who are wealthy will have access to comfortable accommodation and the chances are high that they will have spaces to use such as large gardens, gyms, and swimming pools. Many citizens live on farms or plots. These people can drink coffee on their deck every day in their pyjamas without any significant impact on their lives or daily existence (Kraaijeveld 2021). Wealthy people may be able to live comfortably and independently for longer periods because they can have all kinds of goods and food delivered to their home and will have greater access to online recreation. That is why an investigation indicated that richer people encountered fewer problems with in-home confinement and the restriction of their rights than poorer ones (Bohler-Muller, et al. 2020). However, it should also be mentioned that more prosperous people who live in complexes or estates also do not always have access to outside spaces (Smart and Broadbent 2020).

Individuals and families who are not so well off find themselves in smaller and sometimes very small spaces such as single rooms or have to share accommodation with others (Yamin 2021). In some small shacks ten persons or more may be residing. Others may live in apartment units on the seventh floor. Most of these people’s opportunities for fresh air and movement are extremely limited. In many cases, when nature calls, people have to leave home due to the lack of adequate facilities within the house, it necessitates the individual to relieve themselves outdoors within the view of the community (Smart and Broadbent 2020).

Because of limited space, the chances are greater that poorer and other vulnerable people’s physical health can be harmed. “No jogging. No dog walking. Stay inside” was the definite message from the government. The restriction on exercise had the potential to affect people’s physical health. The absence of physical activity for a period of three weeks can potentially result in various adverse effects, particularly among older individuals and those with existing health conditions (Ten Have 2022). These effects may include muscle loss, damage to the neuromuscular junction, denervation of muscle fibers, insulin resistance, diminished aerobic capacity, weight gain, and the presence of low-grade systemic inflammation. Exercise also has a protective function against various health conditions such as cardiovascular disease, obesity and many others (Adebiyi, et al. 2021). It is especially important for older vulnerable people in order for them to maintain or improve their health.

There is a very high risk for poorer people with limited space who already struggle with psychological and psychiatric problems (depression, anxiety disorders) that their conditions may worsen due to the stay-at-home order (Adebiyi, et al. 2021; Ten Have 2022). Studies made in the United States and Italy point out that citizens’ psychological and psychiatric problems have drastically worsened during confinement (Kraaijeveld 2021). The stay-at-home order in South-Africa was also the cause of psychological and psychiatric illnesses in many citizens and this was proportionally greater among the poorer incarcerated citizens according to one study (Fisher, et al. 2022). It is known that people started to suffer from “lockdown fatigue” early on. Tentative evidence based on Google Trends suggests that, during the enforced lockdown, searches for the words “bored,” “worried,” and “lonely” increased drastically (Brodeur, et al. 2020). In contrast, exercise (as the simple freedom to take a walk around the block) can provide great relief for psychological and psychiatric conditions. People tend to ignore the value of exercise during physical and mental conditions. Physical activity can have particular value for improving physical and psychological conditions (Smart and Broadbent 2020). The result of being confined to the house is that people behave in a more and more sedentary manner. They sit in front of the television, mobile phone and computer for hours. This can lead to a decrease in visual health and a variety of other health conditions (Adebiyi, et al. 2021).

Domestic violence, rape, and child abuse are serious social and health problems in South Africa. Domestic violence means that men, women, and children are physically and psychologically assaulted, with the result that their health is drastically damaged in many cases. The danger of violence at home can begin or increase during forced confinement and, due to the limited space at their disposal, it affected the poorer population disproportionately when compared with wealthier communities where individuals enjoy larger spaces; although, importantly, this fact did not exclude the wealthier communities from the violence (Heywood 2020; Ten Have 2022). In times of major disasters and epidemics, domestic violence has been shown to increase. People literally have nowhere to go and frustrations build up which can lead to violence (Smart and Broadbent 2020). The reality is that the measure of not being allowed to leave the house forced victims of domestic violence to stay with their partner or parents and prevented them from escaping or seeking help. During the stay-at-home order, the government’s domestic violence centre received more than 120,000 calls, which is roughly double the normal number (Adebiyi, et al. 2021; Kell, et al. 2022; Human Rights Watch 2020; Fisher, et al. 2022).

In developing nations, individuals who lack employment or engage in informal and irregular work face a lack of income, support, and food if they are forced to stay at home (Ten Have 2022). A study by Mbatha, et al. (2021, 313) concludes,

This created some challenges for the accessibility of food, as some of the citizens lost their jobs and incomes as they were mandated to stay at home. The overall aspect of the findings reveals that food security in South Africa is being threatened by the sale of unsafe food by the informal sector that mostly supplies the poor. (see also Abrahams, et al. 2022).

It is widely acknowledged that insufficient access to an adequate food supply can greatly compromise individual health. Throughout the course of history, the connection between food and its influence on health has been intricately entwined, capable of either promoting well-being or causing harm (Ten Have 2019).

Injustice, in my opinion, is also demonstrated in existing health programmes (Ten Have 2022). In March and April 2020 alone, the number of AIDS tests dropped by 57 per cent due to the fact that people had to stay in their homes. Because many people were wary of leaving their homes out of fear, it is estimated that the interruption of testing and the availability of chronic medicines led to increased death due to HIV and tuberculosis by 10 to 20 per cent over the subsequent five years. This means that 63,000 more people than usual will die of tuberculosis in the foreseeable period (Smart, et al. 2020).

In summary, preliminary research seems to suggest that the stay-at-home order may have disadvantaged poorer people more than wealthier ones. This directive likely led to large poorer families being forced to live longer in smaller housing, resulting in physical and psychological problems, domestic violence, shortage of healthy food, and an increase in the non-treatment of potential AIDS/HIV and tuberculosis patients. The aim of this study is to assess the stay-at-home order against the global principles of the UDBHR. In light of the aforementioned information, it appears that particularly vulnerable individuals may not have received equal treatment according to the understanding of Article 10. Hence, it is possible to cautiously and tentatively argue that there is reason to question the fulfilment of justice, specifically in terms of equal and fair treatment, as well as the equitable distribution of advantages and disadvantages

The question emerges, which among these two principles carries the greatest weight? The preference of one principle over another is not a simple matter. This weighing process is not an exact science: it sometimes involves an extremely complex process and different information can make the decision weigh in any direction (UNESCO 2008). Article 26 of the UDBHR states that

This Declaration is to be understood as a whole and the principles are to be understood as complementary and interrelated. Each principle is to be considered in the context of the other principles, as appropriate and relevant in the circumstances.

Given this, Kraaijeveld (2021) offers the following heuristic key within the context of the COVID-19 pandemic:

An important principle here is that of the least restrictive means, which holds that public health measures should interfere with the autonomous freedom of individuals to the least possible or necessary extent.

In light of the current difference of opinion on whether the stay-at-home-order curbed the spread of COVID-19 and the heightened sense of belief that the advancement of justice is being questioned, my current view is that justice should weigh heavier than the severe restriction of human freedom. However, this is not the final word on this matter and calls for further and in-depth study.

## Summary

The South African government announced the much-discussed stay-at-home order between March 27, and April 30, 2020, during what was known as lockdown level 5, which meant that citizens were not allowed to leave their homes. The objective of this study is to assess the stay-at-home order against the global principles of the UDBHR. It is deducible that, in reference to the UDBHR, the government possessed the right to curtail individual liberty, thereby not infringing on Article 5 of the UDBHR and therefore, in this context, passes the test of the UDBHR. However, it remains uncertain at present whether the limitation of freedom imposed by the South African stay-at-home order was successful in controlling the spread of COVID-19 and protecting individuals from harm.

Initial investigations also indicate that individuals who are particularly vulnerable may not have received equitable treatment in accordance with the principle outlined in Article 10, therefore, it can be cautiously and modestly argued that the stay-at-home order does not withstand scrutiny when assessed against the UDBHR. Given the continued discussion about the efficacy of limiting freedom to control the spread of COVID-19, and the growing conviction that the advancement of justice is being called into question, the notion of least restriction ought to be considered seriously. Ten Have (2022) is correct in asserting that global bioethics should also seriously consider other principles beyond an almost exclusive focus on limiting individual freedom. The preliminary conclusion is that the potential implementation of the stay-at-home order in the future must be seriously reconsidered.
